# A Case-Series Observation of Sweat Rate Variability in Endurance-Trained Athletes

**DOI:** 10.3390/nu13061807

**Published:** 2021-05-26

**Authors:** JohnEric W. Smith, Marissa L. Bello, Ffion G. Price

**Affiliations:** Department of Kinesiology, Mississippi State University, Starkville, MS 39762, USA; mlb1221@msstate.edu (M.L.B.); fgp13@msstate.edu (F.G.P.)

**Keywords:** hypohydration, hyperhydration, hyponatremia, fluid loss, fluid balance

## Abstract

Adequate fluid replacement during exercise is an important consideration for athletes, however sweat rate (SR) can vary day-to-day. The purpose of this study was to investigate day-to-day variations in SR while performing self-selected exercise sessions to evaluate error in SR estimations in similar temperature conditions. Thirteen endurance-trained athletes completed training sessions in a case-series design 1x/week for a minimum 30 min of running/biking over 24 weeks. Body mass was recorded pre/post-training and corrected for fluid consumption. Data were split into three Wet-Bulb Globe Thermometer (WBGT) conditions: LOW (<10 °C), MOD (10–19.9 °C), HIGH (>20 °C). No significant differences existed in exercise duration, distance, pace, or WBGT for any group (*p* > 0.07). Significant differences in SR variability occurred for all groups, with average differences of: LOW = 0.15 L/h; MOD = 0.14 L/h; HIGH = 0.16 L/h (*p* < 0.05). There were no significant differences in mean SR between LOW-MOD (*p* > 0.9), but significant differences between LOW-HIGH and MOD-HIGH (*p* < 0.03). The assessment of SR can provide useful data for determining hydration strategies. The significant differences in SR within each temperature range indicates a single assessment may not accurately represent an individual’s typical SR even in similar environmental conditions.

## 1. Introduction

The lean tissue of the human body is composed of approximately 73% water [1]. Variations in body fat will result in individual body water levels ranging from ~50–70% of total body mass. This water is critically important for cardiovascular function and thermoregulation. When the body has adequate fluid intake to match fluid losses individuals are considered to be in a state of euhydration. When fluid intake is in excess of fluid loss individuals can become hyperhydrated and when fluid loss exceeds fluid intake individuals become hypohydrated.

Hypohydration is the term used to describe a state of suboptimal body water. At rest, internal factors that influence the body’s water status are mainly body composition, hormonal activity, and sweating [2]. External factors with the greatest influence on body water levels include fluid intake, medications, medical conditions, physical activity, environmental conditions, and clothing [3]. Many athletes, from youth to professional, initiate training in hypohydrated states [4,5,6], and dehydration through sweat loss with insufficient fluid replacement will exacerbate this hypohydrated condition. As a result of hypohydration there is increased cardiovascular strain [7], thermal strain [8], perceived exertion [9], and reduced oxygen and nutrient delivery to the exercising muscle [8,10,11]. Due to these physiological responses to reductions in body water, exercise performance has been shown to be diminished with as little as 2% hypohydration [12,13,14,15,16,17,18].

Hyperhydration is the term used to describe a state of overhydration. At rest excess fluid consumption typically leads to increased urine output allowing for the maintenance of a euhydrated state. In individuals with compromised kidney function, unnecessary increases in fluid intake above fluid loss can lead to fluid retention [19]. Similarly, exercising individuals can experience fluid retention due to the increased actions of antidiuretic hormone and aldosterone upregulation during exercise [20,21]. Studies have demonstrated hyperhydration does not aid in exercise performance or heat tolerance [22,23,24,25,26]. While hyperhydration does not benefit exercise it can be detrimental to health. Uncompensable fluid intake can lead to dilution of electrolytes, particularly sodium, leading to hyponatremia, and if untreated can result in cerebral or pulmonary edema leading to death [8].

The evaporation of sweat can remove ~580 kcal/L and serves as a valuable tool to dissipate heat produced through metabolic processes [27]. As we exercise or perform physical work, metabolic heat production increases to match the increased work output. Sweat rate also increases in an effort to combat the increases in body temperature associated with increased metabolic rate. Unfortunately, sweat that drips from the body and does not evaporate does not provide a significant source of heat loss. In environments with higher humidity, sweat rates can be elevated significantly without fully corresponding to estimated metabolic heat production due to reductions in evaporative cooling as a result of moisture in the air [28]. 

There have been numerous studies exploring average sweat rates and the impact of intensity, duration, environmental conditions, and clothing [29,30]. Typical sweat rates are reported between 0.5–2.0 L/h during activity [31], although due to the large number of variables influencing sweat rate there is significant variability in the sweat rates reported across and within sports. It has been previously reported that about 2% of athletes have sweat rates that can exceed 3 L/h with the highest reported sweat rate during exercise is 5.73 L/h [29]. Within the extremes of the sweat rate range, little to no sweat is produced as a result of conditions such as hypohidrosis and anhidrosis, while hyperhidrosis can lead to extremely high sweat rates [32]. Reporting an average sweat rate when such large ranges exist can result in athletes incorrectly using and applying the information as the foundation for their individual hydration needs. 

The most frequently used method to assess sweat loss in both laboratory and field settings is through pre- and post-exercise body mass changes during exercise. This technique is recommended as a viable method of assessing exercise sweat rate by the American College of Sports Medicine [31] and National Athletic Trainers Association [8]. Limited research has explored the within-subject variability of sweat rate, with day-to-day variation reported to be 5–7% in well-controlled settings. Due to the ease of measuring changes in body mass pre- and post-exercise this technique is commonly recommended to professional and recreational athletes. While athletes have been exposed to this technique, many athletes use it without the full understanding of the controls in place for laboratory-based studies likely resulting in increases in sweat rate variation on a day-to-day basis. Therefore, the purpose of this study was to determine the variability observed in day-to-day sweat rate within endurance trained individuals carrying out regular training without artificially controlled preparation, environmental, and exercise guidelines.

## 2. Materials and Methods

### 2.1. Study Participants

Individuals training as recreational runners and triathletes along with collegiate cross-country runners were recruited from the local area to explore the variability in sweat rate throughout multiple seasons. Data collection trials began in September and concluded in February as a result of COVID-19 restrictions. Thirteen endurance-trained males (*n* = 3) and females (*n* = 10) were included in the present study and were currently running a minimum of 120 min per week for the previous three months. All participants provided written consent prior to participating. This study was approved by the Institutional Review Board at Mississippi State University. 

### 2.2. Experimental Design

Participants completed training sessions once per week for a minimum duration of 30 min in a case-series design. Sessions included either running or biking at a self-selected pace and intensity between 5:30 and 9:30 am. Duration and distance were measured using the athlete’s GPS watches, pace was then calculated from these values. Environmental conditions were recorded using a Wet-Bulb Globe Thermometer (WBGT; QUESTemp 32, 3M, St. Paul, MN, USA) at exercise initiation and every 15 min during the training. Sweat rates were calculated from the change in body mass measured (Defender 3000, OHAUS, Parsippany, NJ, USA) before and after training, with correction for fluid intake. Immediately prior to the initiation of exercise athletes weighed dry exercise clothes and shoes alone, were asked to void their bladder, and then athlete body mass was collected while wearing dry exercise clothes and shoes. To account for sweat trapped by clothes [33], immediately post-exercise athletes’ body mass was collected while wearing exercise clothes and shoes, athletes then changed to allow sweaty clothes and shoes to be weighed alone.

Sweat rate was calculated by:

(1) $$\mathrm{Sweat}\;\mathrm{Rate}=\frac{\left({\mathrm{CBW}}_{\mathrm{PRE}}-{\mathrm{CBW}}_{\mathrm{POST}}\right)+\left(CS_{\mathrm{WET}}-CS_{\mathrm{DRY}}\right)+\left(FB_{\mathrm{PRE}}-FB_{\mathrm{POST}}\right)}{\mathrm{Time}\left(h\right)}$$

where, CBW = clothed body weight (kg); CS = exercise clothing and shoes (kg); FB = food/beverage (kg).

Due to the short duration of exercise no adjustments were made to account for respiratory fluid losses.

Data collection took place outdoors in natural environmental conditions over 7 months except during times of precipitation. The natural environmental conditions were separated into three WBGT ranges: LOW (less than 10 °C), MOD (between 10–20 °C) and HIGH (above 20 °C). 

### 2.3. Statistical Analysis

All data were analyzed using SPSS v26 statistical software (IBM, Armonk, NY, USA). Participant comparisons were included if a minimum of two sessions were completed for LOW, MOD, or HIGH conditions, and the highest and lowest sweat rates were used for analysis, as well as the mean sweat rates. A Shapiro-Wilks test of normality was conducted, there were no outliers, and the significance was above an alpha level of 0.05, therefore the data is normal and is parametric. Not all participants completed multiple training sessions in each range, therefore independent sample T-tests were used for comparisons between WBGT, duration, distance, and pace for each temperature range. Analysis of variance (ANOVA) tests with Tukey’s Honestly Significant Difference (HSD) pairwise comparisons were used to analyze differences in mean sweat rates between temperatures. The participants who completed multiple training sessions in all three temperature ranges (*n* = 4) were included for subsequent analysis. A Friedman’s Rank test was performed to test for differences between temperature ranges. Post-hoc analysis with Wilcoxon Signed-Rank test was conducted with a Bonferroni correction applied. Significance was set a priori at *p* < 0.05.

## 3. Results

Participants displayed no significant change between first and last training session (68.2 ± 14.7 kg vs. 68.4 ± 14.9 kg respectively; *p* = 0.61). Participant sessions were split into WBGT ranges for analysis. There were no significant differences in duration, distance, pace, and WBGT for any of the groups (*p* > 0.07). These data are shown in Table 1.

There were significant differences in sweat rate variability for all groups. LOW WBGTs demonstrated an average difference of 0.15 L/h in sweat rate between highest and lowest recordings (*p* < 0.01). MOD WBGTs showed an average difference of 0.14 L/h in sweat rate (*p* < 0.05). HIGH WBGTs revealed an average difference in sweat rate of 0.16 L/h (*p* < 0.01). Individual sweat rates are shown in Table 2 for each temperature range. Sweat rates for the four participants who completed training sessions within each WBGT range are represented in Table 3.

**Figure 1:**
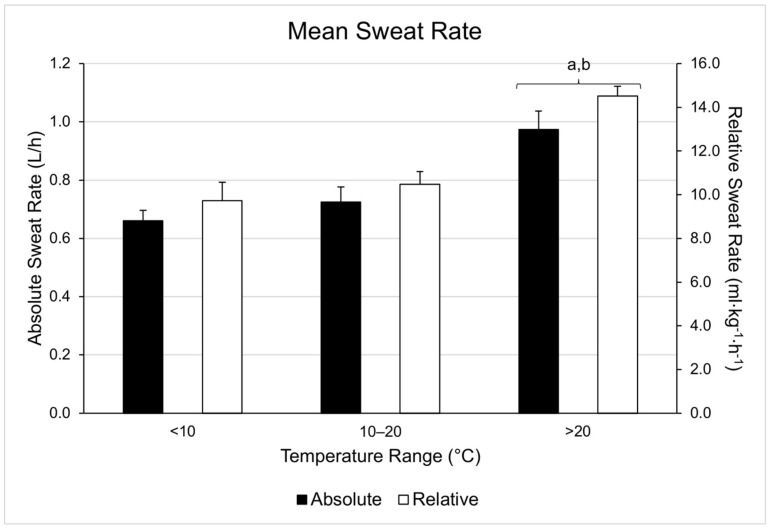
Mean sweat rates (absolute: L/h; relative: mL·kg^−1^·h^−1^) within each temperature range. a = significantly different from LOW, b = significantly different than MOD.

**Figure 2:**
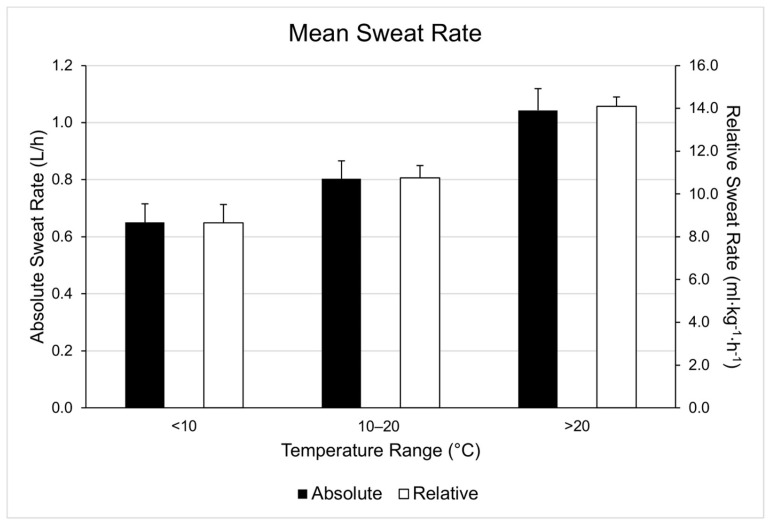
Mean sweat rates (L/h) for four participants that completed training sessions within each temperature range.

Pairwise comparisons revealed no significant differences in sweat rate between LOW and MOD temperatures (*p* > 0.9), but significant sweat rate differences were found between LOW and HIGH (*p* < 0.03) and between MOD and HIGH (*p* < 0.01). These differences are represented in Figure 1. In the four participants who completed sessions in each range, there was a statistically significant difference in mean sweat rate depending on temperature condition, χ^2^(2) = 6.500, *p* = 0.039. Post hoc analysis revealed changes in temperature conditions did not elicit a significant change in mean sweat rate (Low-Mod, *p* = 0.144; Mod-High, *p* = 0.68; Low-High, *p* = 0.68). However it should be noted the differences in mean sweat rate mirrored those seen in the group totals. The differences between temperature ranges are shown in Figure 2.

## 4. Discussion

Hypohydration resulting from the avoidance of fluid ingestion during exercise resulted in a number of publications and position stands in the 1990s promoting fluid ingestion in an effort to prevent related detrimental effects [34]. These positions have been updated as new data are released, moving away from statements such as “…consume the maximal amount that can be tolerated [34].” Initially, athletes used this guideline to unexpected levels, consuming fluid at greater levels than needed to replace fluid losses. This hyperhydration resulted in a number of deaths related to hyponatremia [35]. Due to these unfortunate events, the update to the American College of Sports Medicine in 2007 changed their recommendation to drinking to prevent dehydration and acknowledged the risk of hyponatremia with overdrinking. This position stand recommended the use of body weight changes during exercise to create an individualized hydration plan [31]. The 2016 Position Stand for “Nutrition for Athletic Performance” [36], while stating the prevalence of hypohydration and hypernatremia is thought to be greater, further expounded on the greater risks associated with hyponatremia and again suggested the use of exercise-related body mass changes for the development of an individualized hydration plan. The current study found the variation in day-to-day sweat rate (±7.9–11.7% from the individual’s average in those conditions) is greater than what has previously been reported [37,38].

Hypohydration is a result of inadequate replacement of body water lost mainly through urine, feces, respiration, and sweat at rest. As the degree of hypohydration increases, the subsequent increases in cardiovascular strain [7], thermal strain [8], and perceived exertion [9] are exacerbated. Further, the reduction of oxygen and nutrient delivery to the exercising muscle increase correspondingly as a result of reductions in plasma volume [8,10,11]. Typically these responses do not significantly affect physical activity performance until reaching a threshold of a 2% change in body mass [36]. However, as plasma volume declines, heart rate increases to maintain cardiac output and blood flow to the skin is reduced leading to more rapid elevations in body temperature [8,10].

The ingestion of hypotonic fluids (sports drinks) disproportionately to fluid losses can lead to hyponatremia [39], defined as blood sodium levels below 135 mmol/L with or without the presence of symptoms [40]. While hyperhydration and hyponatremia can occur at rest, in team sports, and during shorter events, they are more commonly seen in endurance and ultra-endurance events. This hyperhydration is largely the result of overzealous efforts to prevent the previously mentioned deleterious effects resulting from hypohydration [40] and lack of exposure to the updated findings and takeaways in the scientific publications on the topic. 

The prevention of hyponatremia and hypohydration are both critically important. It has been suggested that athletes primarily rely on thirst as a means to determine fluid ingestion in relation to exercise-associated fluid losses [41]. Contrary to this position, research has suggested that the reliance on thirst as a mechanism for fluid ingestion can lead to hypohydration, therefore, athletes should approach activity or exercise with an appropriate hydration plan [42]. Recommendations from the American College of Sports Medicine and the National Athletic Trainers Association both recommend the monitoring of body mass changes during exercise as a method to prevent detrimental levels of hypohydration while ensuring hyperhydration is also not occurring [8,31]. Through the simplicity of body weight assessment even recreational athletes can develop an individualized hydration plan.

A number of variables within and outside athlete’s control affect sweat rate. Athletes can often dictate the time of training, their hydration status at the beginning of exercise, clothing, intensity, duration, and some medications. Outside of their control are environmental conditions, hormonal activity, medical conditions, and the action of some medications. During competitive events the variables that are under the athlete’s control are typically reduced as a result of racing regulations and in an effort to remain competitive. Most of our understanding related to the variability of sweat rate in athletes is based on research in which athletes received guidance in preparation and execution. This results in a basic understanding of the physiological responses for comparative purposes but offers limited insight into the variability seen in athletes carrying out training in a more practical and real-world setting.

This study reported relative sweat rates in the HIGH condition (14.5 ± 3.8 mL·kg^−1^·h^−1^) similar to the 15.3 ± 6.8 mL·kg^−1^·h^−1^ sweat rates reported by Baker et al. [29]. However, this study also found higher day-to-day sweat rate variations (±7.9–11.7% from the individuals’ average in those conditions) compared to previous research showing 5–7% variation [37,38]. This data also demonstrates that estimations could differ by as much as 23.4% between the lowest compared to the highest sweat rate. While it would be expected to see increased variation compared to more precisely controlled laboratory conditions, the values observed in this study represent potential day-to-day variability experienced by well-trained athletes in situations without well-defined preparatory guidelines.

The variable most frequently considered when assessing sweat rate is the environment, as sweat rate typically increases with elevations in environmental temperature, radiative heat load, and reductions in wind velocity [43]. Increases in humidity result in reductions in heat transfer from the evaporation of sweat causing sweat remaining on the skin or dripping off the athlete to result in reductions in heat removal. Prolonged exposure to this wet skin and a still, humid environment can lead to the blockage of sweat glands (hidromeiosis) resulting in a reduction in sweat rate [43,44]. Clothing is altered in response to environmental conditions and sporting needs, these changes can influence the sweating response due to the ability or inability to dissipate heat to the surrounding environment as well as the microenvironment created by the clothing shell [3,45,46]. This clothing shell will result in increased humidity around the athlete’s skin inhibiting evaporative cooling [47]. Cooler environmental conditions also lead to athletes increasing the clothing coverage creating a microenvironment inside the clothing shell warmer than the surrounding atmospheric air [47]. The current study found increases in sweat rate in the HIGH environment but similar sweat rate responses to the LOW and MOD conditions. As the athletes were given the freedom to choose exercise apparel it is likely that alterations in athlete clothing in response to LOW conditions resulted in a microenvironment that resulted in similar heat stress and sweating response to the MOD conditions.

As an individual acclimatizes to their training environment the body adapts to be more efficient in its sweating response. As the thermal load was lower in the LOW and MOD conditions, athletes only required a percentage of the sweat rates seen in the HIGH conditions. Adaptations to exercise in hot conditions lead to reductions in sweat sodium concentration, earlier onset of sweating [43], and increases in sweat rate [48,49]. While these adaptations were not assessed in this study, it would be expected that sweat sodium content and maximal sweat rate shifted throughout the study as a result of seasonal variations in environmental conditions and the corresponding sweat rates. 

Another unaccounted-for variable in this study was hydration status at the beginning of exercise. As mentioned previously, hypohydration at levels greater than 2% performance decrements can be seen. Along with the declines in performance when an individual becomes more than 2% hypohydrated [8], they may experience a decline in sweat rate [50], assuming an initial euhydration status. Similarly, most research studies are conducted with defined initial hydration levels as a requirement to participate, while without defined guidelines a majority of athletes begin exercise in a hypohydrated state [8]. As a result of hypohydration the sweating response can be altered resulting in an increased threshold for onset of sweating [51] and reduced sweating sensitivity [52]. This study allowed athletes to arrive at assessments as they would to any other workout or training session. As a result, it is likely a majority of the athletes were hypohydrated at the initiation of exercise which may partially explain the increase in variability reported in this study as compared to previous studies.

Additionally, exercise intensity plays a role in the sweat response, as studies have found higher sweat rates during competition and high-intensity exercise compared with prolonged lower intensity [29,50,53]. This study found similar exercise intensities and durations across the three environmental conditions. As athletes are developing hydration strategies for training and competition attention needs to be given to the variations in intensity in the different settings. As a means to develop a competitive hydration strategy athletes may find it advantageous to perform mock races or training sessions at competitive intensities on a number of occasions within various conditions. While a few athletes in this study that had very little variation in estimates of hourly sweat rate, care needs to be taken in extrapolating sweat rate to longer duration events.

More static factors such as age, sex, tattoos, and medications/medical conditions have also been suggested or shown to also play a role in sweat rate. Adults have higher sweat rates compared to adolescents due to adolescents producing less sweat in the sweat glands [29,54,55]. Males have been shown to have higher absolute sweat rates as compared to females largely driven by higher body mass [29]. When corrected for body mass the increased sweat rate was seen to still be present based on age, but the sex differences disappeared [29]. Some studies have suggested variations in sweat rate in females as a result of menstrual cycle however, a majority of studies reports no difference in sweat rate as a result of menstrual phase [56,57,58]. There is conflicting data in regards to the impact of tattooed skin on sweat rate. Some studies have demonstrated a detrimental effect on sweat rate with tattoos [59,60] while other studies have reported no significant alterations in sweat rate as a result of tattoos [61,62]. Milaria rubia (heat rash), sunburn [63], diabetes mellitus [64], and spinal cord injuries [65,66] highlight a small number (but a large range in severity) of the medical conditions that can influence sweat rate. Likewise the medications that treat many illnesses also can influence sweat rates both positively and negatively [32,67]. While each of these variables can influence sweat rate and should be considered with changes, changes will likely be infrequent but likely result in new sweat rate estimates being needed when changes occur. 

This study was observational leading to significant limitations. This study was conducted as an observation of athletes in the normal activity without influence on their training duration, intensity, initial hydration status, or clothing. The only disruption to the athletes typical training was the requirement for exercise to initiate Monday through Friday between 5:30 and 9:30 am beginning and ending at the laboratory to allow for body mass assessment and changing of clothes. Additionally, only asking athletes to carry out their exercise within these parameters resulted in some athletes not having sessions within all of the environmental (LOW, MOD, HIGH) conditions and resulted in varying numbers of observations within participants due to missed weeks. Finally, the only control for environmental conditions was the lack of trials during periods of precipitation. The use of post exercise clothing mass was used to calculate sweat trapped in clothing or shoes, fluid volume associated with precipitation invalidated this assessment. Environmental variations associated with radiative, convective, humidity, and dry temperature were combined into WBGT for comparisons. Future studies should investigate the variations in sweat rate throughout the various annual seasons within laboratory-controlled assessment conditions mirroring external environmental conditions.

Estimating fluid needs by assessing body mass changes during exercise is a valid technique and a useful tool for athletes [68,69]. However, even following the best practice recommendations [28], a single assessment can result in over and underestimation of true needs. Further this variation can be compounded as sweat rate test findings are extrapolated to longer exercise durations. Compounded errors in sweat rate estimates can place athletes at increased risk of hypohydration and hyponatremia. It should be recommended that body weight changes be assessed across multiple sessions even within the same conditions. The closer an athlete can replicate typical conditions (duration, intensity, clothing, environment, time of day) on multiple occasions the more precise the estimated average sweat loss will represent actual losses and provide more insight into hydration strategies to optimize their training. 

## 5. Conclusions

This data highlights interindividual sweat rate during similar exercise is variable even within similar environmental conditions. Therefore, individuals should repeatedly measure and record sweat rate along with environmental conditions along with exercise intensity to have a reliable estimate of actual sweat rate for intensities and conditions.

## Figures and Tables

**Table 1:** Differences in duration, distance, pace, and Wet-Bulb Globe Thermometer between temperature ranges. (mean ± stdev).

Range	WBGT (°C)	Duration (Minutes)	Running Distance (Miles)	Running Pace (Min/Mile)	Cycling Distance (Miles)	Cycling Velocity (Miles/Hour)
<10 °C	5.7 ± 3.2	42.67 ± 13.33 (*n* = 9)	6.05 ± 4.67 (*n* = 8)	8.40 ± 2.39 (*n* = 8)	21.04 ± 0.03 (*n* = 1)	17.43 ± 0.16 (*n* = 1)
10–20 °C	13.5 ± 2.2	40.63 ± 11.29 (*n* = 6)	5.19 ± 1.82 (*n* = 6)	8.07 ± 1.10 (*n* = 6)		
>20 °C	22.5 ± 1.4	43.29 ± 14.11 (*n* = 11)	6.84 ± 5.60 (*n* = 10)	7.62 ± 1.67 (*n* = 10)	24.41 ± 4.53 (*n* = 1)	19.34 ± 0.83 (*n* = 1)

**Table 2:** Participant sweat rates (L/h) separated into highest, lowest, and mean values. 2A denotes Wet-Bulb Globe Thermometer values below 10 °C, 2B values between 10–20 °C, and 2C values above 20 °C.

**2A.**						
**<10 °C**						
**Subject**	**Low**	**High**	**Mean**	**Relative Mean**	**High-Low**	**Maximal Variation from Mean**
	(L/h)	(L/h)	(L/h)	(mL·kg^−1^·h^−1^)	(L/h)	(%)
1	0.84	0.90	0.87	13.5	0.06	3.4
2	0.43	0.52	0.48	8.5	0.09	9.4
3	0.41	0.56	0.48	7.2	0.15	15.5
4	0.65	0.95	0.84	8.7	0.30	17.8
5	0.97	1.00	0.99	9.6	0.03	1.5
8	0.54	0.61	0.57	9.1	0.07	6.2
9	0.57	0.75	0.66	10.2	0.18	13.7
11	0.39	0.52	0.45	5.2	0.13	14.4
12	0.67	0.90	0.78	12.6	0.23	14.8
**2B.**						
**10–20 °C**						
**Subject**	**Low**	**High**	**Mean**	**Relative Mean**	**High-Low**	**Maximal Variation from Mean**
	(L/h)	(L/h)	(L/h)	(mL·kg^−1^·h^−1^)	(L/h)	(%)
1	1.13	1.23	1.18	11.6	0.10	4.2
2	0.43	0.68	0.60	9.0	0.25	21.0
3	0.58	0.78	0.64	11.9	0.20	15.5
4	0.83	0.84	0.84	10.2	0.01	0.6
6	0.46	0.79	0.57	9.2	0.33	29.1
10	0.49	0.49	0.49	8.5	0.00	0.0
**2C.**						
**>20 °C**						
**Subject**	**Low**	**High**	**Mean**	**Relative Mean**	**High-Low**	**Maximal Variation** **from Mean**
	(L/h)	(L/h)	(L/h)	(mL·kg^−1^·h^−1^)	(L/h)	(%)
1	1.32	1.35	1.33	13.44	0.03	1.2
2	0.81	1.10	0.90	13.9	0.29	16.1
3	0.75	0.96	0.85	16.1	0.21	12.3
4	1.16	1.16	1.16	13.0	0.00	0.0
5	1.62	1.70	1.67	23.0	0.08	2.4
6	0.63	0.81	0.72	12.5	0.18	12.5
7	0.76	0.91	0.82	13.8	0.15	9.2
8	0.64	0.74	0.69	11.9	0.10	7.2
9	1.01	1.22	1.11	17.6	0.21	9.4
10	0.58	0.59	0.59	10.4	0.01	0.9
13	0.79	0.81	0.80	11.6	0.02	0.1

**Table 3:** Participant sweat rates (L/h) for the four participants that completed multiple training sessions within each Wet-Bulb Globe Thermometer range. Sweat rate values shown as highest, lowest, and mean values for each range.

Subject	Temperature	Low	High	Mean
1	<10 °C	0.84 L/h	0.90 L/h	0.87 L/h
	10–20 °C	1.13 L/h	1.23 L/h	1.18 L/h
	>20 °C	1.32 L/h	1.35 L/h	1.33 L/h
2	<10 °C	0.43 L/h	0.52 L/h	0.48 L/h
	10–20 °C	0.43 L/h	0.68 L/h	0.60 L/h
	>20 °C	0.81 L/h	1.10 L/h	0.90 L/h
3	<10 °C	0.41 L/h	0.56 L/h	0.48 L/h
	10–20 °C	0.58 L/h	0.78 L/h	0.64 L/h
	>20 °C	0.75 L/h	0.96 L/h	0.85 L/h
4	<10 °C	0.65 L/h	0.95 L/h	0.84 L/h
	10–20 °C	0.83 L/h	0.84 L/h	0.84 L/h
	>20 °C	1.16 L/h	1.16 L/h	1.16 L/h

## Data Availability

The data presented in this study are available on request from the corresponding author. The data are not publicly available due to participant privacy and confidentiality.
